# Overexpression of *ZePrx* in *Nicotiana tabacum* Affects Lignin Biosynthesis Without Altering Redox Homeostasis

**DOI:** 10.3389/fpls.2020.00900

**Published:** 2020-06-26

**Authors:** Alba García-Ulloa, Laura Sanjurjo, Sara Cimini, Antonio Encina, Romina Martínez-Rubio, Rebeca Bouza, Luis Barral, Graciela Estévez-Pérez, Esther Novo-Uzal, Laura De Gara, Federico Pomar

**Affiliations:** ^1^Departamento de Biología, Centro de Investigaciones Científicas Avanzadas, Universidade da Coruña, A Coruña, Spain; ^2^Unit of Food Science and Human Nutrition, Department of Science and Technology for Humans and the Environment, Campus Bio-Medico University, Rome, Italy; ^3^Área de Fisiología Vegetal, Departamento de Ingeniería y Ciencias Agrarias, Universidad de León, León, Spain; ^4^Grupo de Polímeros, Departamento de Física y Ciencias de la Tierra Escuela Universitaria Politécnica, Universidade da Coruña, Serantes, Ferrol, Spain; ^5^Department of Mathematics, Universidade da Coruña, A Coruña, Spain; ^6^Instituto Gulbenkian de Ciência, Oeiras, Portugal

**Keywords:** class III peroxidases, lignins, syringyl, secondary cell wall, redox homeostasis, RNA-Seq

## Abstract

Class III plant peroxidases (Prxs) are involved in the oxidative polymerization of lignins. Z*innia elegans* Jacq. Basic peroxidase (ZePrx) has been previously characterized as capable of catalyzing this reaction *in vitro* and the role in lignin biosynthesis of several of its *Arabidopsis thaliana* homologous has been previously confirmed. In the present work, *ZePrx* was overexpressed in *Nicotiana tabacum* to further characterize its function *in planta* with particular attention to its involvement in lignin biosynthesis. Since Prxs are known to alter ROS levels by using them as electron acceptor or producing them in their catalytic activity, the impact of this overexpression in redox homeostasis was studied by analyzing the metabolites and enzymes of the ascorbate-glutathione cycle. In relation to the modification induced by *ZePrx* overexpression in lignin composition and cellular metabolism, the carbohydrate composition of the cell wall as well as overall gene expression through RNA-Seq were analyzed. The obtained results indicate that the overexpression of *ZePrx* caused an increase in syringyl lignin in cell wall stems, suggesting that ZePrx is relevant for the oxidation of sinapyl alcohol during lignin biosynthesis, coherently with its S-peroxidase nature. The increase in the glucose content of the cell wall and the reduction of the expression of several genes involved in secondary cell wall biosynthesis suggests the occurrence of a possible compensatory response to maintain cell wall properties. The perturbation of cellular redox homeostasis occurring as a consequence of *ZePrx* overexpression was kept under control by an increase in APX activity and a reduction in ascorbate redox state. In conclusion, our results confirm the role of ZePrx in lignin biosynthesis and highlight that its activity alters cellular pathways putatively aimed at maintaining redox homeostasis.

## Introduction

Peroxidases (EC 1.11.1) are enzymes capable of oxidize many substrates using hydrogen peroxide (H_2_O_2_) as co-substrate (De Gara, 2004; Oliva et al., 2009). Higher plants contain at least four types of peroxidases that exist as multigene families: glutathione peroxidase (EC 1.11.1.9), catalase (EC 1.11.1.7), ascorbate peroxidase (APX, class I peroxidase, EC 1.11.1.11), and class III peroxidases (Prxs, EC 1.11.1.17). Ascorbate peroxidase controls H_2_O_2_ levels by oxidizing ascorbate in the first reaction of the ascorbate (ASC)–glutathione (GSH) cycle, a system that keeps H_2_O_2_ levels under control (De Gara, 2004; Foyer and Noctor, 2011). Prxs are apoplastic and vacuolar plant peroxidases presenting a double catalytic-cycle, which allow them to both consume and produce reactive oxygen species (ROS; Chen and Schopfer, 1999; Zámocky and Obinger, 2010). Several reverse genetic studies, focused on the catalytic properties of these enzymes as well as on their specialized function, demonstrate that Prxs can be involved in a diverse range of functions (reviewed in Shigeto and Tsutstumi, 2016). The diverse functions of Prxs are accomplished through the oxidation of specific molecules such as lignin precursors during lignin polymerization (Sasaki et al., 2004; Gabaldón et al., 2006) and/or through the regulation of specific ROS levels (Kawaoka et al., 2003; Liszkay et al., 2003; Kim et al., 2008; Sundaravelpandian et al., 2013; Peters et al., 2017). The study of Prxs function is challenged by the numerous roles that one isoform can play in plants. Nevertheless, one of the aspects drawing more attention is Prx involvement in lignification (reviewed in Marjamaa et al., 2009).

Lignins, alongside with cellulose and xylan, are the main components of the secondary cell wall (Zhong and Ye, 2015). The biosynthesis of these three macromolecules is finely modulated during plant development and in response to environmental signals in order to accomplish a correct assembly of the secondary cell wall. Several transcription factors (TFs), mainly belonging to NAC and MYB families, are responsible for the regulation of secondary cell wall deposition (Zhong and Ye, 2014; Taylor-Teeples et al., 2015). Thanks to this joint control, changes in the biosynthesis of one of these components can be made up with changes in the biosynthesis of another one, keeping stable the properties of the cell wall (Mélida et al., 2015). Lignins are responsible of making the cell wall hydrophobic and resistant. They are complex and amorphous phenolic polymers, synthesized by oxidative polymerization of three precursors (*p*-coumaryl, coniferyl, and sinapyl alcohols) through a cross-linking reaction initiated by Prxs and laccases in the apoplast (Vanholme et al., 2010). This process increases the strength and rigidity of the cell walls thus with a positive impact on the plant responses to several environmental constraint conditions (Vanholme et al., 2010; Hamann, 2012). Studies performed on *Arabidopsis thaliana* L. mutants for Prx4, Prx52, and Prx72 demonstrated the participation of these enzymes in lignin biosynthesis, especially in the incorporation of syringyl groups to lignins in the interfascicular fibers of the stem (Herrero et al., 2013a; Fernández-Pérez et al., 2014, 2015). These three peroxidases shared features with the *Zinnia elegans* Jacq. basic peroxidase (ZePrx; Herrero et al., 2013b), which has been previously proposed to catalyze the oxidation of monolignols in cells undergoing active lignification such as those of the xylem. It is also able to *in vitro* catalyze lignin precursor oxidative polymerization into molecules enriched in β-*O*-4 links (Ros Barceló et al., 2002; Gabaldón et al., 2005, 2006).

Due to the lack of *ZePrx* mutants or protocols for the stable transformation of *Z. elegans* plants, no further studies had been conducted until now to deepen into the function of ZePrx. The increase of our knowledge on lignification process, cell wall stiffening and cellular redox homeostasis has impact on several applicative aspects such as agronomy yield, biofuel production and plant resilience during environmental constraint conditions. In the present work, *Nicotiana tabacum* L. transgenic plants overexpressing *ZePrx* were obtained and characterized, in order to study the involvement of this enzyme in lignification *in planta* and the redox homeostasis in these overexpressing transgenic plants.

## Materials and Methods

### Plant Material

All plants were kept under controlled conditions on a day/night cycle of 16/8 h at 25/18°C temperature in a growth chamber with white light (150 μmol m^–2^ s^–1^). *Z. elegans* cv. Envy were grown over sterile paper and collected six days after germination. *N. tabacum* plants were grown in sterilized TOPF (Plantaflor) substrate.

### RNA Extraction and cDNA Synthesis

Total RNA from *Z. elegans* hypocotyls and from *N. tabacum* leaves and stems was extracted using Aurum^TM^ Total RNA Mini Kit (Bio-Rad) following the manufacturer’s instructions. For cDNA synthesis with iScript^TM^ cDNA Synthesis Kit (Bio-Rad), 200 ng of RNA were used following the manufacturer’s instructions.

### *ZePrx* Cloning and Expression Vector Construction

ZePrx coding region (AJ880395.1) was amplified by PCR using the ZePrxCLO primers (Table 1) and *Pfu* DNA polymerase (Thermo Fisher Scientific). After purification, the PCR product was cloned into the *Hin*dIII – *Eco*RI site of the MCS of pJIT62 vector using a T4 ligase (Thermo Fisher Scientific). This vector was cloned into chemically competent *Escherichia coli* DH5α. Positive colonies selected after screening on LB medium with 0.01% carbenicillin were confirmed by PCR with ZePrxCLO primers (Table 1). The *Kpn*I-*Xho*I fragment of this vector was purified and introduced into a pGreen II 0179 vector (Hellens et al., 2000a, b), that was cloned into chemically competent *E. coli* XL1 blue cells. Colonies were selected in LB medium supplemented with 0.005% kanamycin, 0.02% X-Gal and 1 mM IPTG and confirmed by PCR with B5 and B3 primers (Table 1). The final vector was sequenced using the primers in Table 1 in a genetic analyzer 3130xl (Applied Biosystems).

**TABLE 1:** Sequences of the different primers used for transgenic lines production.

**Primer**	**Sequence**	**RE**	**Purpose**
ZePrxCLOF	GAGAAAGCTTATGAGTTATCATAAGTCAAGTGGA	*Hin*dIII	Cloning of *ZePrx*, colony screening and sequencing
ZePrxCLOR	GAGAGAATTCTTAACTGGGATTACCGCAAAGAG	*Eco*RI	
ZePrxSEC5	TGTGGTATCGTAAAAGGTGG	–	Sequencing
ZePrxSEC3	ATGTCAACAATCCTGCAACG	–	
B5	GGTAACGCCAGGGTTTTCCCAGT	–	Colony screening and sequencing
B3	CCCGGGCTGCAGGAATTCGAT	–	
ZePrxF	TCTTCCAAGAGGGAACATGG	–	Semiquantitative PCR
ZePrxR	CCGAGTGTGTGAGATCCTGA	–	
NtBTub1F	GAAGGAATGGACGAGATGGA	–	
NtBTub1R	TTCACCTTCGTCATCAGCAG	–	

*Underlined sequences correspond with the restriction enzymes recognition sequences used for *ZePrx* cloning into pJIT60.*

Chemically competent *Agrobacterium tumefaciens* AGL1 (Wise et al., 2006) were transformed simultaneously with pGreen II 0179 and pSoup plasmids (Hellens et al., 2000b). Two types of transformed *A. tumefaciens* were obtained: empty vector lines (EV), transformed with the pGreen II 0179 EV to obtain control plants and plant lines transformed with the pGreen II 0179 vector containing *ZePrx* gene under the control of CaMV 35S promoter to obtain overexpressing (OE) lines. Two independent OE lines were obtained: OE1 and OE2.

### *N. tabacum* Transformation

Surface sterilized seeds of *N. tabacum* cv. Petit Havana (SR1) were sowed on solid half strength MS medium supplemented with 1% sucrose. Leaf fragments of the *N. tabacum* plants were transformed as described in Clemente (2006). Transgenic seeds from self-polinated plants were harvested and stored at 4°C. Resistance analysis was performed to identify T1 lines with only one insertion and T2 homozygous plants through a segregation analysis sowing seeds in a half strength MS medium with hygromycin. Briefly, in order to select transgenic lines with one insertion, we used only T1 lines that showed a 3:1 ratio (resistant:sensible) in the segregation analysis (Bent, 2006; Cosio et al., 2017). Transgenic seeds were harvested and stored at 4°C. Sterilized seeds were sowed in half strength MS solid medium supplemented with 0.1% sucrose and 0.005% hygromycin B. The number of resistant and sensitive plants was determined three weeks after sowing.

### *ZePrx* Expression Levels

Leaves from three weeks-old and stems from six weeks-old EV and OE plants were collected, frozen in liquid nitrogen and stored at −80°C until used. The expression of *ZePrx* (target gene: AJ880395.1) and *NtBTub1* housekeeping gene (reference gene: U91564.2) (Schmidt and Delaney, 2010) were analyzed by using NZYTaq DNA Polymerase (NZYTech) and ZePrx and NtBTub1 primer pairs (Table 1).

### Growth and Development

The stem of *N. tabacum* transgenic plants was measured every three days from the emergence of the first true leave until the opening of the first flower. Its reproductive development was also registered classifying the plants in five categories: vegetative, pre-bloom, budding, flowering and open flower.

### Cell Wall Protein Extraction and Peroxidase Activity

Stems from six and eight weeks-old plants were collected and used fresh for protein extraction. Stems were cut in 0.5 cm long fragments, vacuum infiltrated with 50 mM Tris-acetate 1 M KCl pH 5.0 buffer and centrifuged to collect the intercellular wash fluid (IWF). The IWF was recovered according to Ros Barceló et al. (2002, 2007). This protocol allows the extraction of cell wall components with an irrelevant citoplasmatic contamination (Ros Barceló et al., 2002, 2007; De Pinto and De Gara, 2004). In brief, leaf tissue was homogenized in 50 mM Tris–HCl 1 M KCl 1 mM EDTA pH 7.5 buffer at 4°C. After centrifugation, the supernatant was recovered. All protein samples were dialyzed overnight against their extraction buffer without KCl or EDTA using cellulose membranes with a 14 kDa pore size (Sigma). The protein samples were stored at −20°C until analyzed.

Peroxidase activity was assayed spectrophotometrically at 25°C in a reaction buffer containing 50 mM Tris acetate buffer pH 5.0 for the IWF samples and 50 mM Tris–HCl buffer pH 7.5 for the symplastic ones. The substrates assayed were 1 mM 4-methoxy-α-naftol (4MN) (ε_593_ = 21 μM^–1^ cm^–1^), 50 μM syringaldazine (ε_530_ = 27 μM^–1^ cm^–1^), 100 μM coniferyl alcohol (ε_291_ = 9.75 μM^–1^ cm^–1^) and 100 μM sinapyl alcohol (ε_271_ = 4.14 μM^–1^ cm^–1^). In all the assays 500 μM H_2_O_2_ was used as co-substrate. Total proteins were quantified using Quick Start Bradford (Bio-Rad).

SDS-PAGE gels were prepared according to Laemmli (1970) and proteins were stained with the PlusOne Silver Staining Kit (Pharmacia). Isoelectrofocusing gels were prepared according to Wrigley (1971), migration was performed as described by López-Serrano et al. (2004) and peroxidases were revealed with 4-methoxy-α-naphthol (Ferrer et al., 1990).

### Histochemical Analyses

Cross sections were manually obtained at 1 cm from the base of stem from *N. tabacum* plants. Wiesner staining was performed according to Pomar et al. (2002). For Mäule staining (Strivastava, 1966) the stem cross sections were vacuum infiltrated 5 min in KMnO_4_ 1%, rinsed 2 min in HCl 37%, stained with 0.5 M NH_3_, washed with distilled water and photographed. A preliminary analysis allowed us to define 5–6 weeks after sowing the starting of lignification of the secondary xylem (data not shown).

### Cell Wall Isolation and Characterization

The stem fragments from IWF extraction were homogenized with 50 mM Tris–HCl, 1 M KCl, 1 mM EDTA, pH 7.5 buffer at 4°C. After centrifugation, the pellet was dried at 60°C and, then, milled in a Mini BeadBeater (Biospec Products). The material was washed with 1% Triton X-100 (five times), distilled water (twice), 70% ethanol (once), and 96% ethanol (four times). The pellet was rinsed twice in diethyl ether and dried at 37°C for 48 h (Espiñeira et al., 2011).

Lignin content of the cell walls was determined using the acetyl bromide method. Briefly, 3 mg of cell walls were mixed with 500 μL of acetyl bromide 2.33 M in acetic acid and kept at 70°C for 30 min. NaOH was added to a final concentration of 0.2 N and the final volume was adjusted to 10 mL with acetic acid. Finally, the absorbance at 290 nm was determined and a 20.09 g^–1^ cm^–1^ factor was used for lignin quantification (Iiyama and Wallis, 1988).

Lignin composition was studied by thioacidolysis (Novo-Uzal et al., 2009), adding 0.5 mg of tetracosane to each sample as an internal standard. We used a Thermo Finnigan Trace GC gas chromatograph with a DB-XLB, J&W column (60 m length × 0.25 mm internal diameter) and a Thermo Finnigan Polaris Q mass spectrometer. Mass spectra were recorded at 70 eV. Quantification was performed as described in Yue et al. (2011).

The carbohydrate composition of the cell walls, cellulose, uronic acid sugars and neutral sugars, was determined as described in Mélida et al. (2015). Cell walls were fractioned with KOH extractions (0.1 and 4 N) and total sugar content in these fractions and digestion samples was determined by the phenol-sulfuric method as described in Rebaque et al. (2017). Xyloglucan content was determined on the KOH fractions by iodine sodium sulfate assay (Kooiman, 1960).

Fourier transform infrared spectra (FTIR) of the cell walls were recorded with a Bruker Vector 22 (Bruker Optics).

### Mechanical Tests

The stems of eight weeks-old plants were divided in four segments. Each was subjected to tensile tests performed using a 5566 series Instron universal testing machine (maximum load = 500 N, speed = 0.005–500 mm min^–1^, l = 1135 mm, accuracy = ± 0.01%). All the assays were performed with a displacement speed of mm min^–1^. Tension (σ) and deformation (ε) were used to obtain σ = σ(ε) curves for each segment.

### Analysis of Hydrogen Peroxide and Redox Systems

Hydrogen peroxide was measured in EV and OE plants according to potassium iodide (KI) method (Junglee et al., 2014). Briefly, 0.1 g of leaves were collected and quickly grounded in liquid nitrogen and homogenized with 0.5 ml of 0.1% cold trichloroacetic acid. After centrifugation at maximum speed for 15 min, 0.25 ml of the supernatant were mixed with 0.25 ml of 10 mM potassium phosphate buffer (pH 7.0) and 0.5 ml of KI 1M. The samples were incubated for 15 min at room temperature and then the absorbance at 350 nm was measured. H_2_O_2_ levels of extracts were determined based on a H_2_O_2_ standard curve.

Ascorbate and GSH were extracted as reported in Ferrer et al. (2018). Approximately 0.3 g of leaves were collected from three weeks-old *N. tabacum* plantlets and grinded in liquid nitrogen. The pulverized tissue was homogenized with six volumes of 5% metaphosphoric acid at 4°C. The homogenate was centrifuged at 20,000 *g* for 15 min at 4°C and the supernatant used for the determination of levels and redox states as described in De Pinto et al. (1999). Ascorbate and GSH levels were expressed as nmol mg^–1^ protein.

For the enzymatic activities, leaves from EV and *ZePrx* OE lines were ground in liquid nitrogen and homogenized at 4°C in six volumes of 50 mM Tris–HCl (pH 7.5), added with 0.05% (W/v) cysteine, 0.1% (W/v) BSA and 1 mM ASC. The homogenate was centrifuged at 20,000 *g* for 15 min at 4°C and the supernatant was collected and analyzed by spectrophotometry. The activities of ASC peroxidase (APX) (L-ASC: H_2_O_2_ oxidoreductase, EC 1.11.1.11), DHAR (GSH: dehydroascorbate oxidoreductase, EC 1.8.5.1) and GR (NADPH:GSH disulfide oxidoreductase, EC 1.6.4.2) were determined as previously described (Ferrer et al., 2018).

Non-denaturing-PAGE (protein extraction, gel preparation and staining) was carried out as described in Locato et al. (2008).

### RNA-Seq Analysis

Total RNA from 6 weeks-old stems was extracted using Aurum^TM^ Total RNA Mini Kit (Bio-Rad) following the manufacturer’s instructions. RNA concentration and quality were checked using a Bioanalyzer 2100 (Agilent Technologies). Only samples with a RIN over seven were used.

The RNA-Seq analyses were performed by Macrogen Inc. (Korea Republic). A library was constructed for each biological sample (a total of six) using the TruSeq RNA Sample Prep v2 (Illumina) according to the manufacturer’s instructions. The paired-end sequencing was conducted in an Illumina HiSeq 2500 sequencer with the TruSeq SBS v4 (Illumina) kit.

Data quality was assessed with the software FastaQC v0.11.5. The sequences were trimmed using the SLIDINGWINDOW option of Trimmomatic 0.32 (Bolger et al., 2014). Trimmed sequences were aligned to the reference genome (Edwards et al., 2017) with the software HISAT2 v2.0.5 (Kim et al., 2015) and the transcripts were assembled with StringTie v1.3.3b (Pertea et al., 2015). The abundance of each transcript was determined with the –e option of StringTie (Pertea et al., 2016).

### rt-qPCR

One microgram of RNA was used for cDNA synthesis with iScript^TM^ cDNA Synthesis Kit (Bio-Rad), following the manufacturer’s instructions. The amplification was performed with a FastStart SYBR Green Master mix (Roche). Specific primers at 0.3 μM concentration and 125 ng of cDNA were used for rt-qPCR reaction under the following conditions: 10 min at 95°C, 40 cycles of 30 s at 95°C, 30 s at 55°C, 1 min at 72°C and a final extension of 5 min at 72°C. The analyzed genes and the primers used are depicted in Table 2.

**TABLE 2:** Genes analyzed through qPCR.

**Gene**	**ID**	**Primer**	**Sequence**	**Efficiency (%)**
PAL	Nitab4.5_0000582g0180	NtPALF	GGGCATGGTCCACTAACACT	93
		NtPALR	CTTGCTCACAGCGGTTGATA	
MYB15	Nitab4.5_0006658g0010	NtMYB15F	GCTGGTGCCCATAGTACCTC	92
		NtMYB15R	TGCGAGTTCTCTTGCTGATG	
CesA4	Nitab4.5_0002389g0020	NtCesA4F	CTCATCCCACCAACCACTCT	90
		NtCesA4R	AAGAGAGGACCCCATGAACC	
GT43	Nitab4.5_0000669g0220	NtGT43F	AATTGAGGGATTTGGGACAT	93
		NtGT43R	ATGCCACCCCATAACTTCAG	
β-Tub1	U91564.2	NtBTub1F	GAAGGAATGGACGAGATGGA	100
		NtBTub1R	TTCACCTTCGTCATCAGCAG	
Actin	AB158612	NtActF	GGCATTGCAGATCGTATGAG	97
		NtActR	CCTTGATCTTCATGCTGCTG	

*ID, identification code in the annotation of *N. tabacum* genome (Edwards et al., 2017) or accession in the NCBI database. β-Tub1 (Schmidt and Delaney, 2010) and actin (Duwadi et al., 2015) were used as reference genes.*

### Statistical Analysis

At least three biological replicates for each analysis have been considered. Statistical analysis was performed using SPSS v17.0. Levene test was used to check the homogeneity of variances. ANOVA test was performed to check differences among OE and EV lines. Kruskal–Wallis test was the chosen alternative when ANOVA data requirements were not met. Finally, Tukey or Dunnet *post hoc* tests were used to check for homogenous subsets. In peroxidase activity assays we used the moment of sowing as a random factor, since the effect of this variable was found to be significant.

For FTIR analysis, 10 biological replicates were analyzed. The spectra were baselined corrected and normalized with the OPUS 5.5 (Bruker) software. SPSS v17.0 was used for principal component analysis and the R v3.5.1 package to perform a FANOVA with the FANOVA and fda.usc functions.

The RNA-Seq analysis of differentially expressed genes (DEG) was performed using R Bioconductor after transforming and normalizing the abundance values with the preprocessCore’ R library. Only genes with an absolute value of fold change over two and a p-value under 0.05 were consider as DEGs. These genes were grouped according to their expression profile using a hierarchical cluster (Euclidean distance and whole linkage). We performed a Gene Ontology (GO) Enrichment Analysis on all the genes with a *p*-value under 0.05 using the PANTHER enrichment analysis tool (Mi et al., 2019). A Fisher test was performed using a Benjamini-Hochberg False Discovery Rate correction with a 0.05 cut-off value. We used de REVIGO tool in order to eliminate redundant categories (Supek et al., 2011). We analyzed separately upregulated and downregulated genes.

## Results

### Characterization of Overexpressing *ZePrx* Transgenic Lines

#### Overexpressing *ZePrx* Transformed Lines Accumulated High Level of an Active Protein

*N. tabacum* plants were transformed with the ZePrx coding sequence under the control of the strong and constitutive CaMV35S promoter and eleven stable independent transformed lines were obtained. The expression of *ZePrx* was confirmed both in stems and leaves. Semi-quantitative rt-PCR analysis performed on stem and leaves revealed that *ZePrx* gene expression was higher in most of the OE compared to EV lines where the *ZePrx* gene expression was undetectable (Supplementary Figures S1A,B). Two OE lines (named 2.15 and 7.2) and two EV lines (10.3 and 15.14) were selected and further characterized. In the following results we present the mean value for EV lines, as they showed a similar behavior.

Since ZePrx has been reported to catalyze the last step of lignin biosynthesis (Gabaldón et al., 2005), the impact of its overexpression was firstly investigated in the stem, an organ in which the lignification is pivotal, especially during its secondary growth. At this purpose, two developmental stages have been considered for subsequent analysis based on direct observation of the plants growing in our experimental conditions: six weeks-old plants, when stems are starting their secondary growth, and eight weeks-old plants, when secondary growth has already occurred and stems are widely lignified.

**FIGURE 1:**
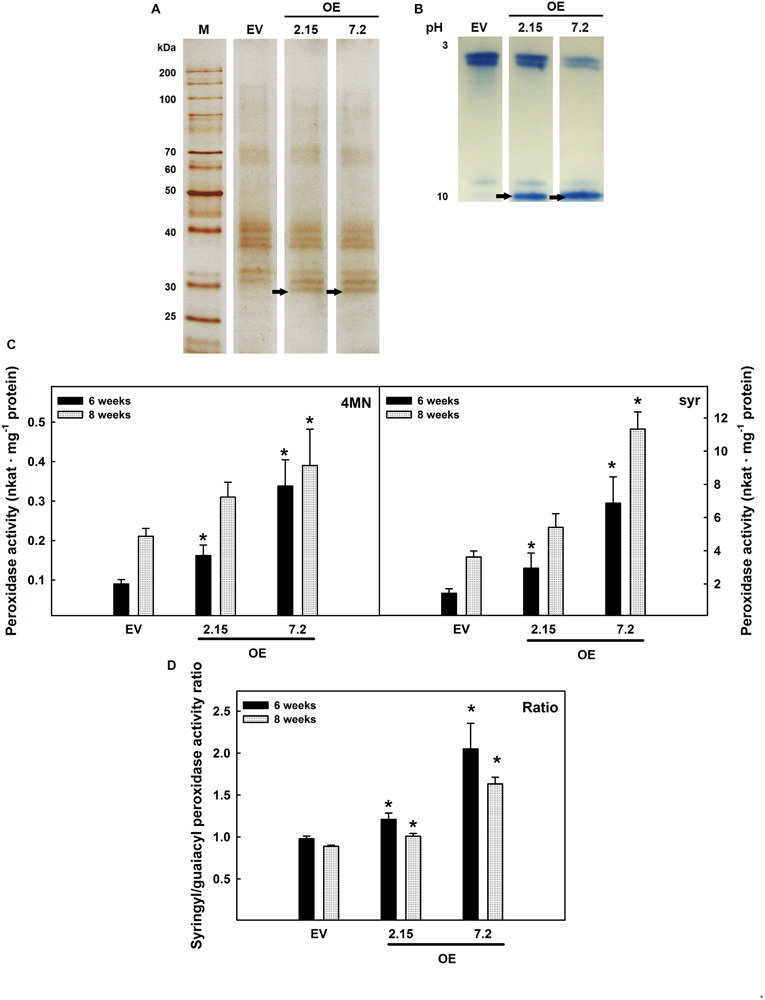
ZePrx detection and peroxidase activity in the intercellular wash fluid from stems. **(A)** SDS-PAGE gels and **(B)** Isoelectrofocusing gels of proteins from the apoplast of the stems of 6 weeks-old *N. tabacum* transgenic plants. EV, empty vector; OE, *ZePrx* overexpressing lines. Arrows signal the extra band corresponding to the presence of ZePrx. **(C)** Peroxidase activity on the apoplastic fluid of six and eight weeks-old stems of *N. tabacum* transgenic plants. Total peroxidase activity was determined using 4-methoxy-α-naphthol (4MN) and syringyl-peroxidase activity, with syringaldazine (syr). **(D)** Ratio S/G, between the peroxidase activity measured with sinapyl alcohol (syringyl) and coniferyl alcohol (guaiacyl). The figure shows the mean and standard error for each of the lines. EV, empty vector; OE, *ZePrx* overexpressing lines; Con Alc, coniferyl alcohol; Con Ald, coniferaldehyd; Sin Alc, sinapyl alcohol; Sin Ald, sinapaldehyde. Asterisks (*) indicate which OE lines were found to be significant different from the control in a Dunnet *post hoc* test at 0.05 level of significance. *n* = 3 for each line. EV is the mean of four EV lines (*n* = 12).

ZePrx protein presence in stems collected from EV and OE lines at six weeks-old developmental stage was confirmed by SDS-PAGE and isoelectrofocusing. Protein separation by SDS-PAGE showed the presence of an additional band, of approximately 30 kDa, in the apoplastic fluid of OE lines (Figure 1A). Eight weeks-old plants showed the same protein and isoenzyme pattern (data not shown). The presence of active ZePrx protein in OE lines was confirmed by isoelectrofocusing stained for Prx activity (Figure 1B). An isoelectric point close to 10 that corresponds to basic peroxidases characterized this additional band. The size and the isoelectric point of this additional protein detected in the OE lines were coherent to those previously determined for ZePrx (Gabaldón et al., 2005).

#### *ZePrx* Overexpression Caused an Increase in Stem Peroxidase Activity but Did Not Have any Effects on Development

The peroxidase activity in the IWF obtained from stem of six and eight weeks-old plants was measured using four different substrates: 4MN and syringaldazine, synthetic substrates, as well as coniferyl and sinapyl alcohol, two lignin building blocks. The peroxidase activity measured using 4MN substrate (which measures the total peroxidase activity) and syringaldazine (which measures the so-called S-peroxidase activity) was significantly increased in the IWF of both OE lines at six-weeks-old and at eight weeks-old stems in OE 7.2 line (Figures 1C,D). In both cases, the peroxidase activity measured in OE 7.2 line was higher than that measured in 2.15 OE line (Figure 1C). This behavior agrees with the ZePrx overexpression level detected in the two transgenic lines. The ratio between the peroxidase activity measured using sinapyl alcohol (syringyl activity) and coniferyl alcohol (guaiacyl activity) was also analyzed in order to verify if the overexpression of a ZePrx affected it. This ratio, referred as sinapyl/guaiacyl (S/G ratio) gives information about the type of lignin precursors that the peroxidases are able to oxidize, since not all the Prxs are capable of oxidizing sinapyl alcohol (Sasaki et al., 2004). Both OE lines showed a higher S/G activity ratio when compared to EV lines (Figure 1D).

**FIGURE 2:**
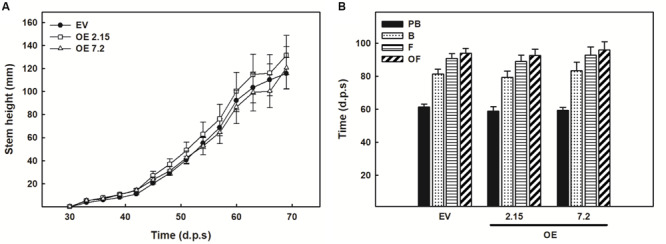
Development of transformed plants. **(A)** Evolution of the stem height of the *N. tabacum* transgenic plants. **(B)** Time that used each line to reach each of the four developmental stages defined: pre-bloom (PB), blooming (B), flowering (F), and open flower (OF). The figure shows the mean and standard error for each of the variables. d.p.s., days post sowing; EV, empty vector; OE, *ZePrx* overexpressing lines. *n*(EV) = 39, *n*(OE 2.15) = 21 and *n*(OE 7.2) = 18.

OE and EV lines did not show any differences in stem height during its vegetative growth (Figure 2A). Moreover, no differences in the time needed to reach different stages of floral development were evident between OE and EV lines (Figure 2B).

#### Redox Homeostasis in OE Lines Required the Involvement of APX

Since it is well known that the diverse peroxidase activities can facilitate opposite reactions in plants, such as generation and scavenging of ROS thus affecting plant homeostatic capacity, the effects of ZePrx overexpression on antioxidative systems and H_2_O_2_ content were investigated.

**FIGURE 3:**
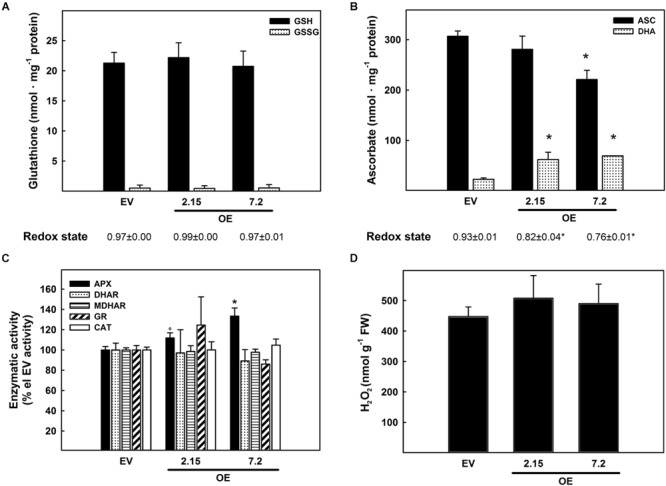
Ascorbate-glutathione cycle in leaf samples from three weeks-old plants. **(A)** Content of reduced glutathione (GSH), oxidized glutathione (GSSG), and redox state expressed as GSH/(GSH + GSSG) ratio. **(B)** Content of reduced ascorbate (ASC), oxidized ascorbate (dehydroascorbate DHA), and redox state expressed as ASC/(ASC + DHA) ratio. **(C)** Relative activity of the ascorbate-glutathione cycle enzymes. The following values are relative to the EV line: ascorbate peroxidase (APX) 6.67 nmol ASC ox min^–1^ g^–1^ FW; dehydroascorbate reductase (DHAR) 0.63 nmol DHA red min^–1^ g^–1^ FW; monodehydroascorbate reductase (MDHAR) 1.43 nmol NADH ox min^–1^ g^–1^ FW; glutathione reductase (GR) 0.11 nmol NADPH ox min^–1^ g^–1^ FW; catalase (CAT) 2.49 nmol H_2_O_2_ dism min^–1^ g^–1^ FW. **(D)** Hydrogen peroxide quantification with KI. The figure shows the mean and standard error for each of the lines. EV, empty vector; OE, *ZePrx* overexpressing lines. Asterisks (*) indicate which OE line were found to be significant different from the control in a Tukey *post hoc* test at 0.05 level of significance. Asterisks (°) indicate which OE line were found to be significant different from the control in a Tukey *post hoc* test at 0.1 level of significance. *n* = 3 for each line for the ASC-GLU experiments and *n* = 5 for each line in the hydrogen peroxide experiment. EV is a mean of two EV lines (n_ASC–GLU_ = 6; n_perox_ = 10).

The effects of *ZePrx* overexpression on the ASC-GSH cycle was analyzed by measuring the content of these two antioxidants, their relative redox state and the activities of the enzymes involved in this cycle. The activity of catalase (CAT) was also measured. All these parameters were monitored in the leaves (see Materials and Methods), which also overexpressed *ZePrx* (Supplementary Figure S1C) and where ASC-GSH cycle plays a pivotal role for maintaining cellular homeostasis (De Gara et al., 2010). Leaves did not show any statistically significant difference in the content of the reduced (GSH) and oxidized forms (GSSG) of glutathione between the analyzed lines (Figure 3A). The content of total ascorbate pool was also not changed between EV and OE lines but an increase in the content of the oxidized form, dehydroascorbate (DHA), and a decrease in the content of the reduced form, ASC, was observed in the two OE lines compared to EV lines. Therefore, a decrease in the ascorbate redox state occurred in OE plants (Figure 3B). Consistently, the activity of APX, the first enzyme of the ASC-GSH cycle controlling H_2_O_2_ levels by using ascorbate as reducing agent, was significantly higher in the two OE lines than in the EV lines (Figure 3C). None of the other ASC-GSH cycle enzymes as well as CAT were significantly altered in the transformed lines (Figure 3C).

To check whether the overexpressed ZePrx was, itself, responsible for the increased ASC oxidation in the OE lines, the pattern of the peroxidases able to use ASC as electron donor was analyzed by non-denaturing PAGE. The absence of an extra band in the APX isoenzyme profile of OE lines compared with EV ones (Supplementary Figure S2), confirmed that the rise in ASC oxidation and APX activity was not directly due to ZePrx activity. To get deeper insights into redox impairment putatively due to *ZePrx* overexpression, the H_2_O_2_ content was also measured in the leaves. The increased observed in H_2_O_2_ content in OE lines was not statistically significant (Figure 3D).

### *ZePrx* Overexpression Caused Few Changes in Cell Wall Composition

**FIGURE 4:**
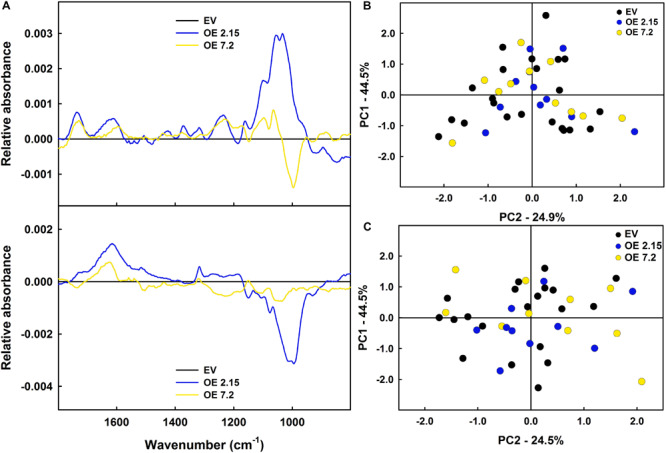
FTIR of stem cell walls. **(A)** Differential spectra for six weeks-old plants. **(B)** Differential spectral for eight weeks-old plants. FANOVA tests did not found differences among lines in any region of the spectra neither at six (*p*-value_CFF_ = 0.6840; *p*-value_VE_ = 0.6280) or eight weeks-old plants (*p*-value_CFF_ = 0.3405; *p*-value_VE_ = 0.2630). **(C)** Factorial punctuations of six weeks-old plants. **(D)** Factorial punctuations of eight weeks-old plants. The figure shows the mean for each of the lines. In figures **(C,D)** the explained variance of each component is displayed in the axes. EV, empty vector; OE, *ZePrx* overexpressing lines. *n* = 10 for each line. EV is a mean of two EV lines (*n* = 20).

As a first approach to study the changes induced in cell wall composition by *ZePrx* overexpression, FTIR analysis was used. This technique offers global information of cell wall composition by an infrared-spectrum (see section “Materials and Methods”). The obtained data did not show relevant differences in OE and EV spectra when statistically analyzed (Figures 4A–C). However, since small changes in specific cell wall components are hard to be detected by FTIR analysis, lignin deposition and carbohydrates composition of cell wall were analyzed in EV and OE lines. The total lignin content was measured by acetyl bromide method. EV and OE lines showed no statistically significant differences in lignin content in six weeks-old stems, where the lignin content was about 70 μg mg^–1^ cell wall, and in eight weeks-old stems, with lignin values around 87 μg mg^–1^ cell wall (Table 3). The absence of overall changes in lignin deposition was also confirmed through histochemical analysis that did not show differences between EV and OE lines on the amount and/or distribution of cinnamaldehydes (Wiesner staining) and syringyl lignins (Mäule staining). These latest tests also showed that vascular structure was not affected by the overexpression of *ZePrx* (Supplementary Figure S3).

**TABLE 3:** Lignin quantification and composition of the stems of *N. tabacum* transgenic plants.

**Line**	**Lignin (μg mg^–1^ cell wall)**	**Monomeric composition (%)**				**S/G ratio**
		**Con Alc**	**Con Ald**	**Sin Alc**	**Sin Ald**	
**6 weeks-old stems**						
EV	71.51 ± 6.49	45.57 ± 1.29*	2.09 ± 0.20	49.17 ± 1.43*	2.93 ± 0.24	1.11 ± 0.06*
OE 2.15	71.17 ± 4.85	45.74 ± 1.87*	2.20 ± 0.31	48.73 ± 2.71*	3.33 ± 0.79	1.09 ± 0.09*
OE 7.2	69.93 ± 1.40	43.94 ± 0.98*	2.08 ± 0.25	50.91 ± 1.43*	3.08 ± 0.20	1.18 ± 0.06*
**8 weeks-old stems**						
EV	87.40 ± 4.53	42.38 ± 0.28*	1.53 ± 0.06	53.66 ± 0.34*	2.43 ± 0.44	1.28 ± 0.01*
OE 2.15	86.43 ± 5.63	42.09 ± 0.39*	1.55 ± 0.07	53.89 ± 0.41*	2.47 ± 0.16	1.29 ± 0.02*
OE 7.2	87.98 ± 12.16	39.78 ± 1.00*	1.47 ± 0.07	56.49 ± 0.79*	2.26 ± 0.26	1.43 ± 0.06*

Lignin quantification through acetyl bromide method and monomeric composition determined by thioacidolysis. The mean and standard error values are provided. EV, empty vector; OE, *ZePrx* overexpressing lines; Con Alc, coniferyl alcohol; Con Ald, coniferaldehyde; Sin Alc, sinapyl alcohol; Sin Ald, sinapaldehyde. Asterisks (*) indicate which OE lines were found to be significantly different from the control in a Dunnet *post hoc* test at 0.05 level of significance. *n* = 3 for each line. EV is a mean of 2 EV lines (*n* = 6).

Lignin composition was more in depth studied by thioacidolysis in order to determine the percentage of lignin derived from coniferyl alcohol, coniferaldehyde, sinapyl alcohol and sinapaldehyde in six and eight weeks-old stems. In addition, the S/G ratio was calculated to check for differences in the proportion of syringyl to coniferyl units in the lignins characterizing OE and EV lines (Table 3). While there were no changes in lignin composition in six weeks-old plants, an increase in lignin derived from sinapyl alcohol was found at 8 weeks-old in the OE 7.2 line and, consequently, an increase in S/G ratio (Table 3).

**FIGURE 5:**
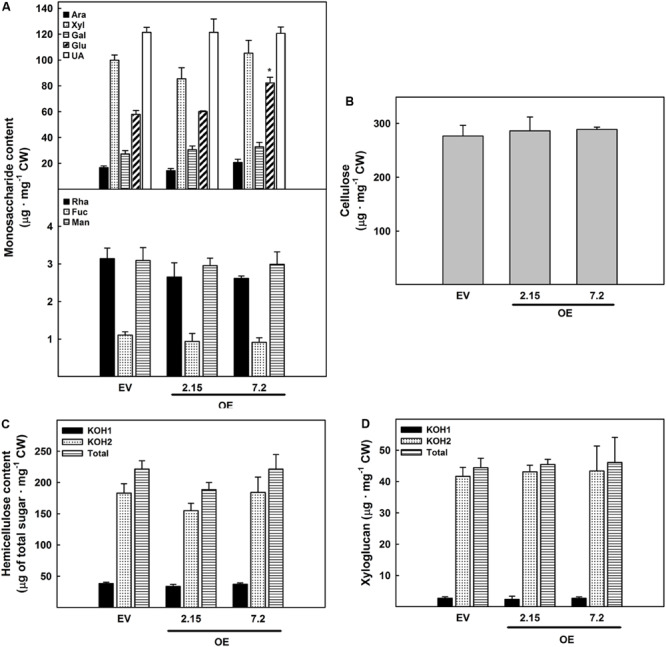
Carbohydrate composition of eight weeks-old stems cell walls. **(A)** Monosaccharide composition (Ara, arabinose; Xyl, xylose; Gal, galactose; Glu, glucose; UA, uronic acid; Rha, rhamnose; Fuc, fucose; and Man, mannose). **(B)** Cellulose content. **(C)** Total sugar content of the two different hemicellulose fractions of the cell wall (KOH1: fraction extracted with KOH 0.1N and KOH2: fraction extracted with KOH 4N). **(D)** Xyloglucan content of the different hemicellulose fractions of the cell wall. The figure shows the mean and standard error for each of the lines. EV, empty vector; OE, *ZePrx* overexpressing lines. Asterisks (*) indicate which OE lines were found to be significant different from the control in a Dunnet *post hoc* test at 0.05 level of significance. *n* = 3 for each line and *n* = 6 for total sugar content in the hemicellulose fractions. EV is a mean of two EV lines (*n* = 6).

The analysis of the cell wall was extended to carbohydrate composition in OE and EV lines stems of eight weeks-old plants, a developmental stage in which there were significant changes in lignin composition in OE 7.2 line. Most of the monosaccharides present in cell walls showed no differences among lines. However, there was a significant increase in the glucose content in the OE 7.2 line (Figure 5A). In cell wall, glucose is present in two main polysaccharides: cellulose and hemicelluloses. Therefore, the content of these two polysaccharides was analyzed. Neither cellulose content (Figure 5B) nor hemicellulose content and distribution (Figure 5C) revealed significant differences among EV and OE lines. Since xyloglucan is the hemicellulose with the highest glucose content, xyloglucan content in the hemicellulose fractions was also measured but no differences were observed (Figure 5D).

### Cell Wall Properties Were Not Affected by *ZePrx* Overexpression

**FIGURE 6:**
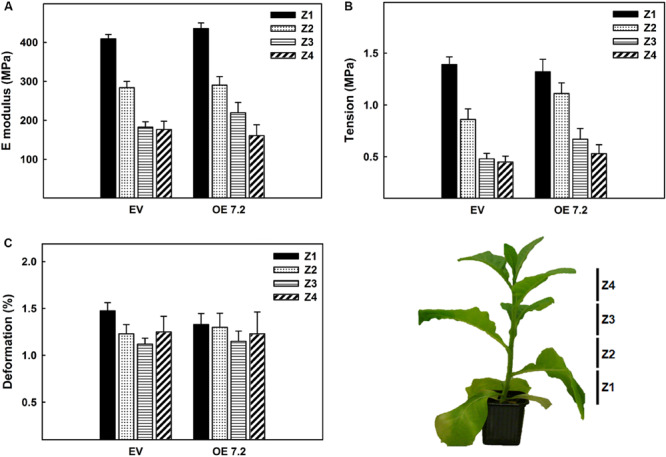
Mechanical tests of eight weeks-old stems determined in a tension test. **(A)** E modulus. **(B)** Maximal tension. **(C)** Relative deformation of the stem. The figure shows the mean for each of the lines. EV, empty vector; OE, *ZePrx* overexpressing lines. Asterisks (*) indicate which OE lines were found to be significant different from the control in a Dunnet *post hoc* test at 0.05 level of significance. *n* = 5 for each line. EV is a mean of two EV lines (*n* = 10).

Changes in cell wall composition can alter cell wall mechanical properties. For this reason, a tensile test measuring elasticity (E-modulus), tensile strength and deformation, was performed in eight weeks-old stems of the EV and OE 7.2 lines (Figures 6A–C). For these experiments the OE 7.2 line was selected since it has a higher peroxidase activity compared with OE 2.5 line and also a significant modification of the cell wall composition. No differences were observed in the E-modulus, the tension nor the deformation that the stems can bear between EV and OE 7.2 lines (Figures 6A–C).

### *ZePrx* Overexpression Caused Changes in the Secondary Cell Wall Biosynthesis Network

**FIGURE 7:**
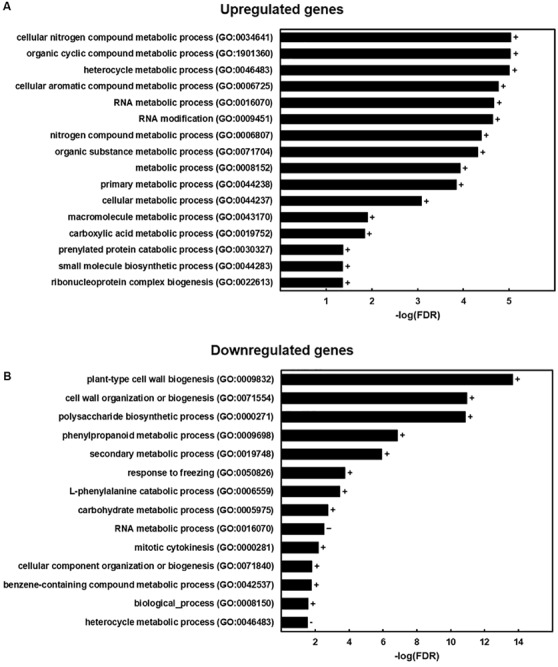
GO enrichment analysis. GO terms that were more represented in the genes with a significant change in expression of the RNA-Seq analysis (*p*-value < 0.05) that in *N. tabacum* genome. **(A)** Results for the group of upregulated genes (427 total genes, but 297 were assigned with a GO category and included in the analysis). **(B)** Results for the group of downregulated genes (354 total genes, but 274 were assigned with a GO category and included in the analysis). The figure shows the -log(FDR) for each category obtained with a Benjamini-Hochberg FDR correction. Signs next to the bars indicate if a category was overrepresented (+) or underrepresented (–) in the sample.

An RNA-Seq analysis was performed on six weeks-old stems in order to further deepen in the impact of *ZePrx* overexpression on the overall plant metabolism. The OE 7.2 line was again selected for this analysis among with the EV 15.14 line. 781 genes showed a significant change in fc (*p*-value < 0.05 and a fc ≥ |1|). 354 of these genes were downregulated and 427 upregulated (Supplementary Table S1). An enrichment analysis has been performed in order to identify the GO categories overrepresented in both groups (Figure 7). These results showed that OE 7.2 line had an upregulation of genes related to RNA modification and different metabolic processes (Figure 7A) and a downregulation of genes related to cell wall biosynthesis, both carbohydrates and lignin (Figure 7B).

**FIGURE 8:**
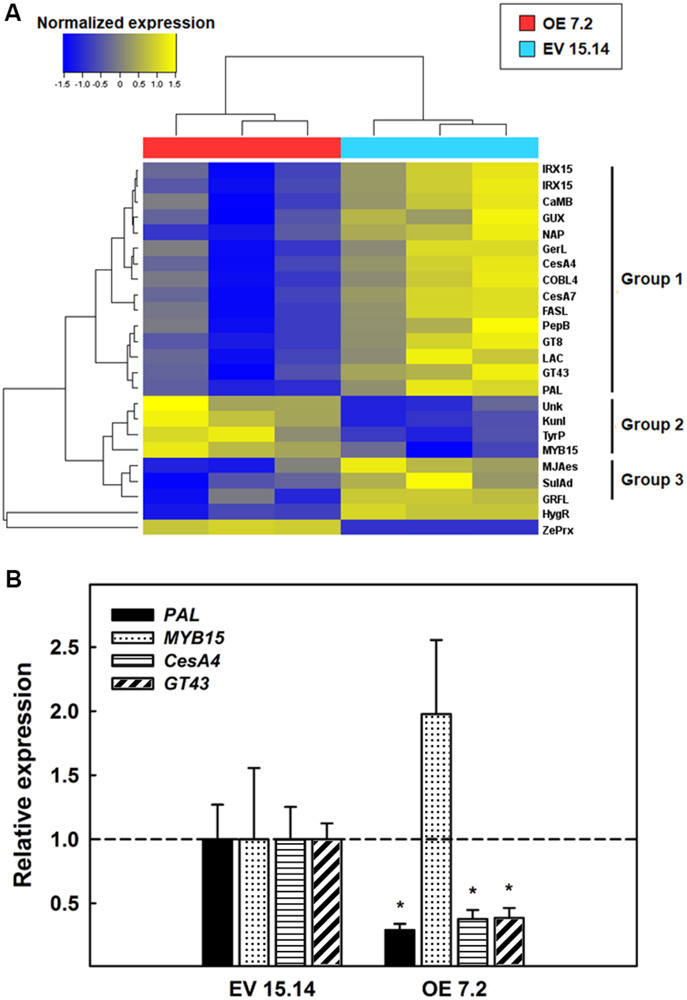
RNA-Seq of six weeks-old stems. **(A)** DEG clustered according to its expression patterned in the two lines analyzed (EV 15.14 and OE 7.2). DEGs with a *p*-value < 0.05 and a fc > 2 are included. Genes are detailed in Supplementary Table S2. **(B)** Relative expression determined by qPCR of four DEGs. The figure shows the mean for each of the lines. EV, empty vector; OE, *ZePrx* overexpressing lines. Asterisks (*) indicate which OE lines were found to be significant different from the control in a Dunnet *post hoc* test at 0.05 level of significance. *n* = 3 for each line.

We enlarge the analysis by including DEGs with a bigger change in their expression (fc > |2|). From this analysis have emerged 22 DEGs (18 downregulated and 4 upregulated) (Figure 8A and Supplementary Table S2). Through a hierarchical clustering, it was possible to divide the 22 DEGs into three groups (Figure 8A). The first group includes genes that code for proteins involved in secondary cell wall biosynthesis. All these genes were repressed in the OE line. A phenylalanine ammonio lyase (PAL) and a laccase, both involved in lignins biosynthesis; two cellulose synthase isoforms (CesA4 and CesA7) and a COBRA-like protein (COBL4), responsible for secondary cell wall cellulose biosynthesis; two glycosyltransferases (GT43 and GT8) and three proteins with a DUF579 domain (two isoforms of a IRX15L protein and a GUX protein), contributing to xylan biosynthesis; an arabinogalactan protein fasciclin-like that keeps cell wall structure; and four proteins related to cell death (a germin-like protein, a CaM-binding protein, a nepentesin-like protein and a peptidoglycan binding protein with a LysM domain). The other two groups contained genes involved in signaling. Group 2 contained four genes that were induced in the OE 7.2 line: a MYB TF (*MYB15*), a Kunitz-like protease inhibitor, a tyrosine phosphatase and a protein of unknown function. Meanwhile group 3 contained three genes that were repressed in the OE 7.2 line: a methyl jasmonate esterase, a sulfate adinilitransferase ATP-sulfurilase and Zinc-finger protein GRF-like. These groups, especially the first one, showed a correspondence with the GO enrichment analysis for the genes with a significant fc ≥ |1|, since genes involved in cell wall biosynthesis were especially represented.

In order to confirm the information derived from RNA-Seq analysis, the expression of four genes was analyzed by qPCR: *PAL* (lignin), *CesA4* (cellulose), *GT43* (xylan), and *MYB15* (Figure 8B). The first three genes were selected in order to corroborate the changes in expression of genes directly involved in the biosynthesis of the main components of secondary cell wall and *MYB15*, since it is a TF belonging to one of the main families that regulate secondary cell wall biosynthesis. Consistently with the RNA-Seq analysis, the expression of the first three genes, involved in secondary cell wall biosynthesis, was strongly reduced in OE lines compared to EV lines. On the other hand, the expression of *MYB15* was induced in OE lines in comparison with EV lines in accordance with the RNA-Seq analysis (Figure 8B).

## Discussion

Prxs are classical secretory peroxidases involved in numerous biological processes in plants being one isoform possibly involved in more than one function (Cosio and Dunand, 2009; Shigeto and Tsutstumi, 2016). This is the case of AtPrx33 and AtPrx34, involved in plant defense and root elongation (Shigeto and Tsutstumi, 2016). Other example is *swpa4*, a sweet potato peroxidase that, when overexpressed in tobacco, is capable of increasing lignin biosynthesis and tolerance to biotic and abiotic stimuli (Kim et al., 2008). The use of expression studies, biochemical characterization and mutants or transgenic plants has been very useful to assign a precise function to a specific Prx (Cosio and Dunand, 2009). Plant transformation has provided numerous evidences of the *in planta* function of various isoenzymes (El Mansouri et al., 1999; Kawaoka et al., 2003; Kim et al., 2008; Herrero et al., 2013a; Fernández-Pérez et al., 2014, 2015; Cosio et al., 2017; Wu et al., 2017).

ZePrx belongs to class III peroxidase and it is considered responsible of lignin polymerization in *Z. elegans* since its expression in the stem is temporally correlated with its lignification (Ros Barceló et al., 2002). Moreover, this enzyme is capable of oxidizing *in vitro* the three main lignin precursors (*p*-coumaryl, coniferyl, and sinapyl alcohols) to polymers similar to lignins (Gabaldón et al., 2006). The promoter of this enzyme has multiple motifs that respond to signals usually involved in the regulation of vascular development and lignification (Gutiérrez et al., 2009; López Núñez-Flores et al., 2010), backing its role in lignin biosynthesis. In the present work, heterologous overexpression of ZePrx in *N. tabacum* plants has been used to further characterize the function of this enzyme.

Transgenic OE plants showed a higher expression of *ZePrx* compared to EV lines. The presence of an active ZePrx in *N. tabacum* OE lines is confirmed by the identification of an extra band of 30 kDa with an isolelectric point of 10, consistently with the characteristics previously determined for ZePrx (Gabaldón et al., 2005) and an increase in the peroxidase catalytic activity. The shift toward a higher capability of oxidizing sinapyl alcohol, given by the increase in S/G activity ratio, is coherent with the overexpression of a S-peroxidase (Gabaldón et al., 2005). Indeed, ZePrx has five characteristic motifs in its sequence, which determine a wider catalytic site compared to the so-called guaiacyl or G-peroxidases. As a result, they can accommodate larger substrates such as sinapyl alcohol or syringaldazine (Ros Barceló et al., 2007). Under optimal growing conditions, OE transgenic plants are not phenotypically altered, in terms of plant growth and development. These results agree with those of Fernández-Pérez et al. (2014) and Cosio et al. (2017). In the first case, *A. thaliana* knock-out mutants for *AtPrx52*, which codifies a ZePrx orthologous involved in lignin polymerization, did not show any growth or development changes (Fernández-Pérez et al., 2014). In the second one, the authors observed that *AtPrx17*, codifying for another peroxidase involved in lignification, caused notable changes in development when suppressed, but mild ones or none when overexpressed (Cosio et al., 2017).

Prxs are able to produce H_2_O_2_, besides using it as substrate in lignin polymerization (Kim et al., 2008). Indeed, these enzymes undergoes a peroxidative cycle, which consumes H_2_O_2_ by oxidizing different compounds, as well as a hydroxylic and an oxidative cycle through which they produce different types of ROS (Chen and Schopfer, 1999). H_2_O_2_ is considered the main ROS acting in cells, due to its relatively long half-life and its capability to cross biological membranes through aquaporins (Wang et al., 2020). Recently, mechanisms for extracellular H_2_O_2_ perception and integration with internal cues have been suggested to be pivotal for sensing the outside stress conditions (Wu et al., 2020). H_2_O_2_ modulation is crucial, since it is a signaling or damaging molecule, depending on concentration, timing and localization of its production as well as the presence or other signals specifically generated by stressing conditions (De Gara et al., 2010; Noctor et al., 2018). In our case non-significant increase in H_2_O_2_ was observed in OE lines. However, this could be the consequence of the increase in APX activity and ASC oxidation observed in both OE lines. The maintenance of an overall redox homeostasis is also confirmed by RNA-Seq analysis, since no genes included in the redox category have been identified as differentially expressed between OE and EV lines. The interaction between H_2_O_2_-producing peroxidases and APX has been already reported in literature as determinant in various physiological processes, first among all in stress defense responses. Peters et al., found that *Sporisorium scitamineum* (smut pathogen) induces an early modulation of the production and scavenging of ROS during defense responses in resistant sugarcane. In particular, in the resistant genotype infected by *S. scitamineum*, the expression of a protein belonging to class III peroxidases seems to be positively related to an increase in APX activity aimed at reducing intracellular H_2_O_2_ increase (Peters et al., 2017).

Several studies focused on the Prxs involved in lignin biosynthesis showed that its overexpression causes an increase in lignin content (El Mansouri et al., 1999; Kim et al., 2008; Cosio et al., 2017; Wu et al., 2017). However, the overexpression of *ZePrx* in *N. tabacum* plants does not seem to cause a lignin accumulation at higher levels than EV plants, when analyzed by histochemical staining or the acetyl bromide method. These results contrast with previous studies in which mutants of *A. thaliana* lacking the ZePrx orthologous AtPrx4, AtPrx52, and AtPrx72, presented a reduction in lignin deposition (Herrero et al., 2013a; Fernández-Pérez et al., 2014, 2015). Nevertheless, it should be considered that lignin biosynthesis is a major carbon sink for the plant and has a high energy cost (Amthor, 2003). Plants have evolved a complex control system over lignin biosynthesis in order to avoid unnecessary deposition of lignin thus limiting the carbon flux into the lignin biosynthetic pathway through the control of the expression of different key enzymes (Anterola et al., 2002; Richet et al., 2011). Furthermore, some studies have pointed out that lignin precursors are not stored in large quantities in the cell, but transported to the apoplast to become part of lignins as soon as they are synthesized (Anterola et al., 1999; Smith et al., 2013). Since peroxidases are involved in the last step of lignin biosynthesis (Vanholme et al., 2010), lignins precursor availability is a limiting factor influencing the quantity of lignin that plants generate. In this context, an increase in the activity of enzymes involved in the oxidative polymerization, such as ZePrx, will not result in a higher amount of lignins biosynthesis when precursors are scarce. Instead, in the case of mutants with a reduced expression of peroxidases involved in lignin polymerization, the peroxidase activity becomes insufficient for lignin biosynthesis, causing a reduction of total lignins (Herrero et al., 2013a; Fernández-Pérez et al., 2014, 2015; Cosio et al., 2017). RNA-Seq results also showed that the OE line 7.2 had a reduced expression of two genes involved in lignin biosynthesis a PAL and a laccase (Vanholme et al., 2010). Therefore, the plant seems to respond to the expression of an additional peroxidase by limiting the production of monolignols (decrease in PAL) and its incorporation to the lignin polymer (decrease in laccase), thus avoiding an unnecessary increase in lignin deposition due to *ZePrx* overexpression. This does not occur in OE line 2.15 in which only a smaller increase in ZePrx activity was observed in comparison with OE line 7.2. The increase in peroxidase activity occurring in OE 2.15 line seems not to be sufficient to induce a change in lignin composition. Therefore, the two analyzed transgenic lines, 2.15 and 7.2 OE lines, differ from the EV line with respect to a higher peroxidase activity, a higher Syringyl/Guaiacyl peroxidase activity ratio and a higher ASC content and ascorbate peroxidase activity. However, the OE 7.2 line presents a more marked increase in peroxidase activity and a stronger increase of Syringyl/Guaiacyl peroxidase activity ratio compared to the 2.15 line. The change in the peroxidase activity observed at six weeks developmental stage in the 7.2 OE line could be considered not sufficient to cause variation in lignin composition. On the contrary, at eight weeks, developmental stage in which the increase in peroxidase activity was stronger, an interesting variation in lignin composition was observed, further supporting a dose dependent effect of the peroxidase activity on lignification process.

It is worth noting that mutants of *A. thaliana*, lacking the ZePrx orthologous showed a reduced S/G ratio (Herrero et al., 2013a; Fernández-Pérez et al., 2014, 2015). Consistently, in our *N. tabacum* plants overexpressing *ZePrx* the increase in S-peroxidase activity promotes a higher incorporation of syringyl units within lignins thus causing an increase in S/G ratio. This effect seems to be dose dependent, since it increased in the OE lines during time, following the increase of Prx activity. Therefore, the overexpression of *ZePrx* determine a change in lignin composition, without increasing its content. This dose dependent effect, has been also previously observed in similar studies in which mutant lines with a higher peroxidase activity showed a higher modification of lignins content (Elfstrand et al., 2001, 2002; Kim et al., 2008). Elfstrand and coworkers, demonstrate that the overexpression of a peroxidase in *Norway spruce* resulted in an enrichment in coniferaldehyde groups, without affecting to the total lignin quantity. Additionally, in this case the change in lignin composition has effects on lignin structure, since syringyl units are only involved in β-*O*-4 links and this links generate a linear lignin polymer (Sasaki et al., 2004). Lignins with such a structure contribute to cell walls being less elastic (higher E-modulus) that the ones with guaiacyl-type lignins (Koehler et al., 2006). In our case, despite the increase in S/G ratio, the increase in E-modulus was not statistically significant. This might be due to other compensatory alteration on the cell wall. The analysis of cell wall polysaccharides composition only showed an increase in glucose content in OE 7.2 line compared to EV lines. Nevertheless, this increase could not be explained by changes in cellulose, hemicellulose or xylan contents, which are the main storage of glucose in the cell wall. It is interesting to note that glucose determination was performed by degrading cell wall carbohydrates with trifluoroacetic acid (TFA). This method is more efficient in degrading the amorphous cellulose fraction than the crystalline one (Foster et al., 2010). Therefore, the increase in glucose content could be caused by the presence in the cell wall of a higher proportion of amorphous cellulose, without any change in the whole amount of cellulose in the cell wall. This is relevant since cellulose crystallinity also contributes to cell wall elasticity. An increase in crystalline cellulose, that is predominant in the secondary cell wall, increases cell wall stiffness, while an increase in the amorphous one reduces it (Gibson, 2012). This change would compensate the effect on the E-modulus of the raise of syringyl groups in lignin, thus keeping unaltered the elasticity properties of the cell wall. Thus, the change in the cellulose structure would compensate the change in lignin composition, avoiding changes in the mechanical properties of the stem. In accordance with this hypothesis, Mélida et al. (2015) demonstrated that changes in one cell wall component result in compensatory changes in other cell wall components. This hypothesis is also supported by the gene expression analysis. Among the downregulated genes three are involved in the biosynthesis of crystalline cellulose: *CesA4*, *CesA7*, and *COBL4*. The first two are isoforms of cellulose synthase involved in the biosynthesis of highly ordered cellulose microfibrils (McFarlane et al., 2014). The latter is a COBRA-like protein, which is necessary to assemble the crystalline microfibrils of cellulose (Persson et al., 2005; Liu et al., 2013). Therefore, these changes in gene expression, together with the increase in the amorphous cellulose, can explain the increase in glucose observed in the OE 7.2 line after TFA cell wall degradation. Consistently, mutations in the *CesA4* gene in rice caused a reduction in cellulose crystallinity, and an increase in cell wall saccharification (Li et al., 2018).

**FIGURE 9:**
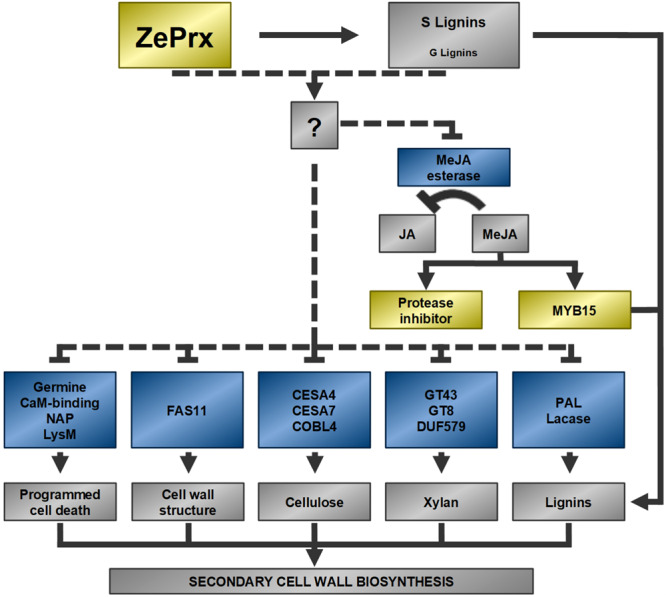
Possible functional relationships between DEGs. Dotted lines represent proposed relationships. Arrowheads represent a positive relationship and blunt ends, a negative one. Blue: repressed genes in the OE line, yellow: induced genes in the OE line, gray: metabolic effects of the described genes, unknown factors and mechanisms. Methyl jasmonate (MeJA); jasmonate (JA); MYB transcription factor 15 (*MYB15*); a nepentesin-like protein (NAP); a peptidoglycan binding protein with a LysM domain (LysM); two cellulose synthase isoforms (CESA4, CESA7); a COBRA-like protein (COBL4); two glycosyltransferases (GT43, GT8); protein with a DUF579 domain (DUF579); phenylalanine ammonio lyase (PAL).

Therefore, *ZePrx* overexpression seems to trigger a compensatory response in the cell wall biosynthesis. Figure 9 shows a possible explanation of gene expression reorganization induced by *ZePrx* overexpression, based on RNA-Seq analysis. Besides genes involved in secondary cell wall biosynthesis and programed cell death which share the same expression pattern, the expression of *ZePrx* also affected the expression of genes involved in signaling. The alteration of TFs of MYB family is of particular interest since this family has a major role in controlling secondary cell wall formation. Specifically, this MYB15 TF is homologous to *AtMYB15*, which has been related to lignin biosynthesis in constraint situations like exposure to cold (Agarwal et al., 2006). In addition, this gene has been reported to be induced by methyl jasmonate (Almagro et al., 2014), which could be accumulate in the OE 7.2 plants as a result of the reduced expression of a methyl jasmonate esterase observed in these plants. This is also the case for some protease inhibitors (Wu et al., 2008), that share with *MYB15* the same gene expression pattern (Figures 8A, 9). Therefore, the alteration of the expression of these genes could contribute to reduce changes in the mechanical properties of the stem putatively induced by the increase in syringyl lignins caused by *ZePrx* overexpression. Nevertheless, further research is needed to fully understand the role of its genes in cell wall homeostasis.

In conclusion, our data suggests that ZePrx participates in lignification *in planta* by altering cell wall composition, in particular the S/G ratio. In the absence of other external stimuli, such as biotic or abiotic stress, the OE lines seem to be able to overcome changes in cell wall mechanical properties by altering the metabolic pathways controlling cell wall structure as well. They were also able to avoid changes in redox homeostasis by modulating APX activity and ASC oxidation. Further research is needed to fully understand the whole function of ZePrx *in planta*, and in particular, its interconnections with redox systems and its possible involvement in stress defense responses.

## Data Availability Statement

The datasets presented in this study can be found in online repositories. The names of the repository/repositories and accession number(s) can be found below: https://www.ncbi.nlm.nih.gov/sra/PRJNA607100.

## Author Contributions

AG-U carried out the experiments, participated in the study, contributed to design and drafted the manuscript. LS designed and participated in the transformation experiments. SC contributed to design the study, to analyze the data and to write the manuscript. AE and RM-R coordinated and performed the carbohydrates experiments. RB and LB designed and performed the tensile tests. GE-P statistically analyzed the FTIR data. LD coordinated the redox experiments and reviewed the manuscript. EN-U contributed to designed the study, coordinated the experiments on lignin composition and helped to draft the manuscript. FP designed the study, coordinated the experiments concerning lignin composition and reviewed the manuscript. All authors contributed to the article and approved the submitted version.

## Conflict of Interest

The authors declare that the research was conducted in the absence of any commercial or financial relationships that could be construed as a potential conflict of interest.
