# Supplementary material for: Overexpression of ZePrx in Nicotiana tabacum Affects Lignin Biosynthesis Without Altering Redox Homeostasis
Source: Front Plant Sci. 2020 Jun 26;11:900. doi: 10.3389/fpls.2020.00900 (PMC7333733; doi:10.3389/fpls.2020.00900)
Supplement: Supplementary file 2 [file Table_2.docx]

| **Table S2**. Differentially expressed genes (DEGs) in RNA-Seq (\|fc\| > 2, p-value < 0.05) in the OE 7.2 sample when compared when the EV 15.14 in six weeks-old stems. The ID makes reference to Figure 8A. The mean *fc* and standard error values are provided. N = 3 for each line. | | | |
| --- | --- | --- | --- |
| ID | Code | Description | *Fc* |
| - Group 1 - | | | |
| IRX15 | Nitab4.5_0000604g0080 | Unknown function protein DUF579 (IRX15L) | -2,02 ± 0,27 |
| IRX15 | Nitab4.5_0002003g0010 | Unknown function protein DUF579 (IRX15L) | -2,17 ± 0,27 |
| CaMB | Nitab4.5_0003077g0050 | Calmodulin binding protein | -2,24 ± 0,38 |
| GUX | Nitab4.5_0007693g0030 | Unknown function protein DUF579 (GUX) | -2,13 ± 0,34 |
| NAP | Nitab4.5_0000496g0150 | Aspartic-endopetidase nepentesin I (NAP) | -2,19 ± 0,22 |
| GerL | Nitab4.5_0001783g0090 | Germine-like protein RmlC-like jelly roll fold | -2,45 ± 0,43 |
| CesA4 | Nitab4.5_0002389g0020 | Cellulose synthase (CesA4) | -2,04 ± 0,28 |
| COBL4 | Nitab4.5_0000939g0030 | COBRA-like protein (COBL4) | -2,20 ± 0,37 |
| CesA7 | Nitab4.5_0002611g0020 | Cellulose synthase (CesA7) | -2,10 ± 0,28 |
| FASL | Nitab4.5_0000101g0040 | Arabinogalactan protein fasciclin-like | -2,13 ± 0,33 |
| PepB | Nitab4.5_0000794g0030 | Peptydoglycan binding protein | -2,09 ± 0,38 |
| GT8 | Nitab4.5_0002229g0130 | Glycosiltransferase familty 8 (GT8) | -2,07 ± 0,33 |
| LAC | Nitab4.5_0000278g0210 | Lacase | -2,82 ± 0,45 |
| GT43 | Nitab4.5_0000669g0220 | Glycosiltransferase family 43 (GT43) | -2,18 ± 0,32 |
| PAL | Nitab4.5_0000582g0180 | Phenylalanine ammonio lyase (PAL) | -2,12 ± 0,26 |
| - Group 2 - | | | |
| Unk | Nitab4.5_0001105g0220 | Unknown protein | 2,19 ± 0,31 |
| KunI | Nitab4.5_0000023g0480 | Kunitz-like protease inhibitor | 2,42 ± 0,23 |
| TyrP | Nitab4.5_0000130g0150 | Tyrosine phosphatase | 2,40 ± 0,29 |
| MYB15 | Nitab4.5_0006658g0010 | MYB transcription factor (MYB15) | 2,13 ± 0,30 |
| - Group 3 - | | | |
| MJAes | Nitab4.5_0000617g0190 | Methyl jasmonate esterase | -2,07 ± 0,25 |
| SulAd | Nitab4.5_0000977g0040 | Sulfate adenilitlransferase ATP-sulfurilase | -2,41 ± 0,40 |
| GRFL | Nitab4.5_0000835g0090 | Zinc-finger protein GRF-like | -3,17 ± 0,40 |
| - Inserted genes - | | | |
| HygR | - | Hygromicin B fosfotransferase (HygR) | -4,88 ± 0,25 |
| ZePrx | - | Basic peroxidase from Zinnia elegans (ZePrx) | - |
