# Supplementary figures and images for: Overexpression of ZePrx in Nicotiana tabacum Affects Lignin Biosynthesis Without Altering Redox Homeostasis
Source: Front Plant Sci. 2020 Jun 26;11:900. doi: 10.3389/fpls.2020.00900 (PMC7333733; doi:10.3389/fpls.2020.00900)

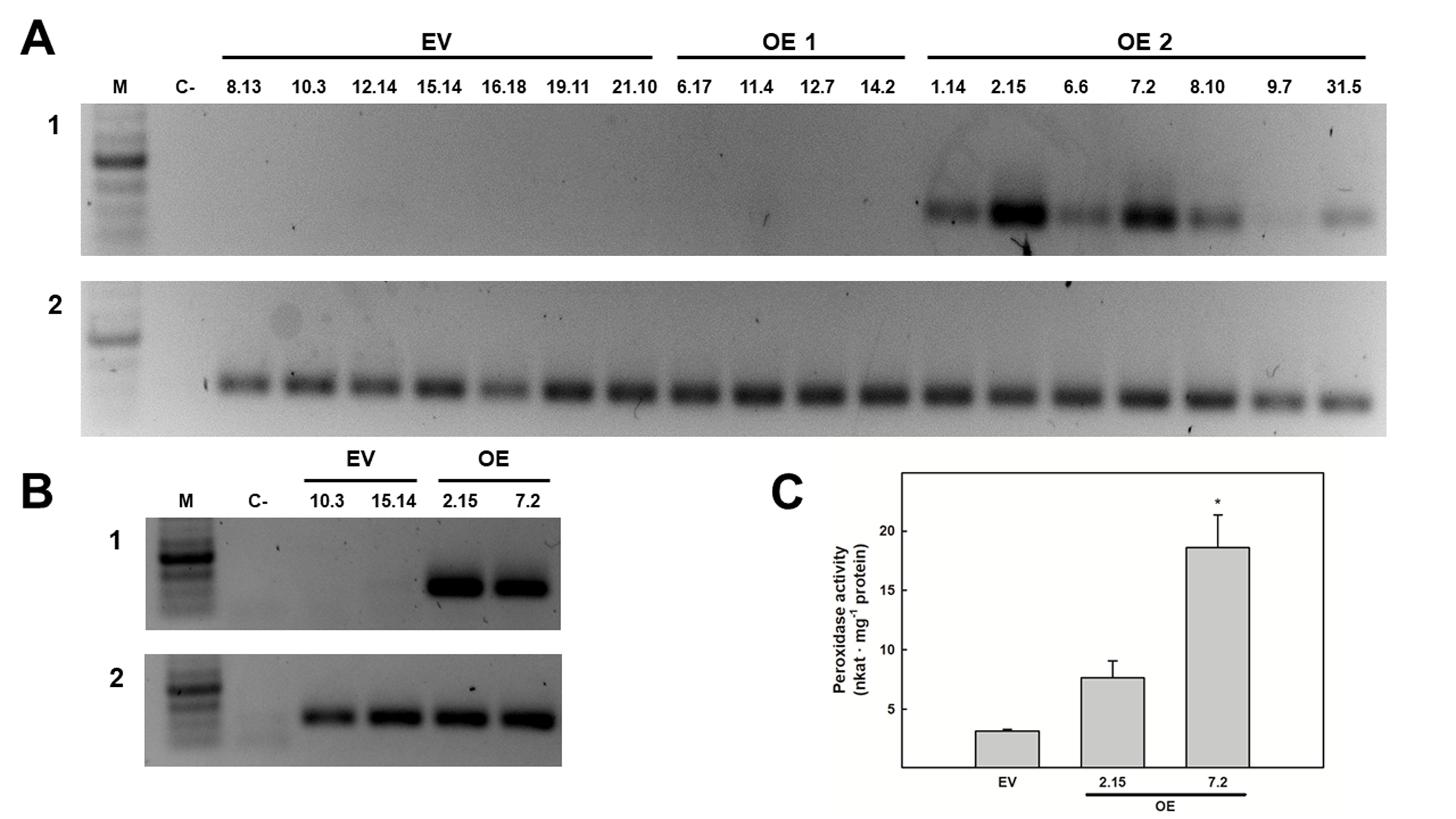

Supplement: Supplementary file 3 [file Image_1.TIF]

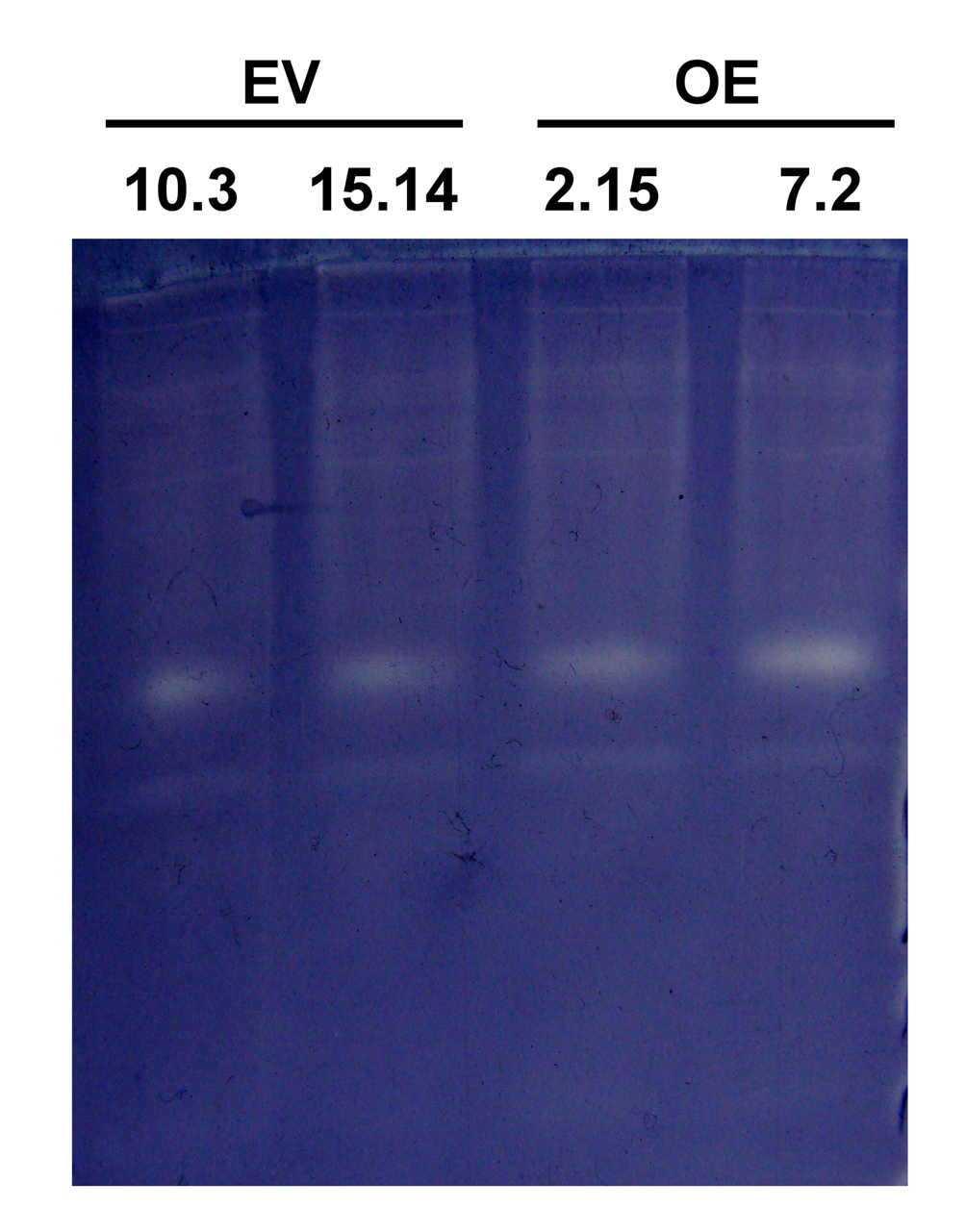

Supplement: Supplementary file 4 [file Image_2.TIF]

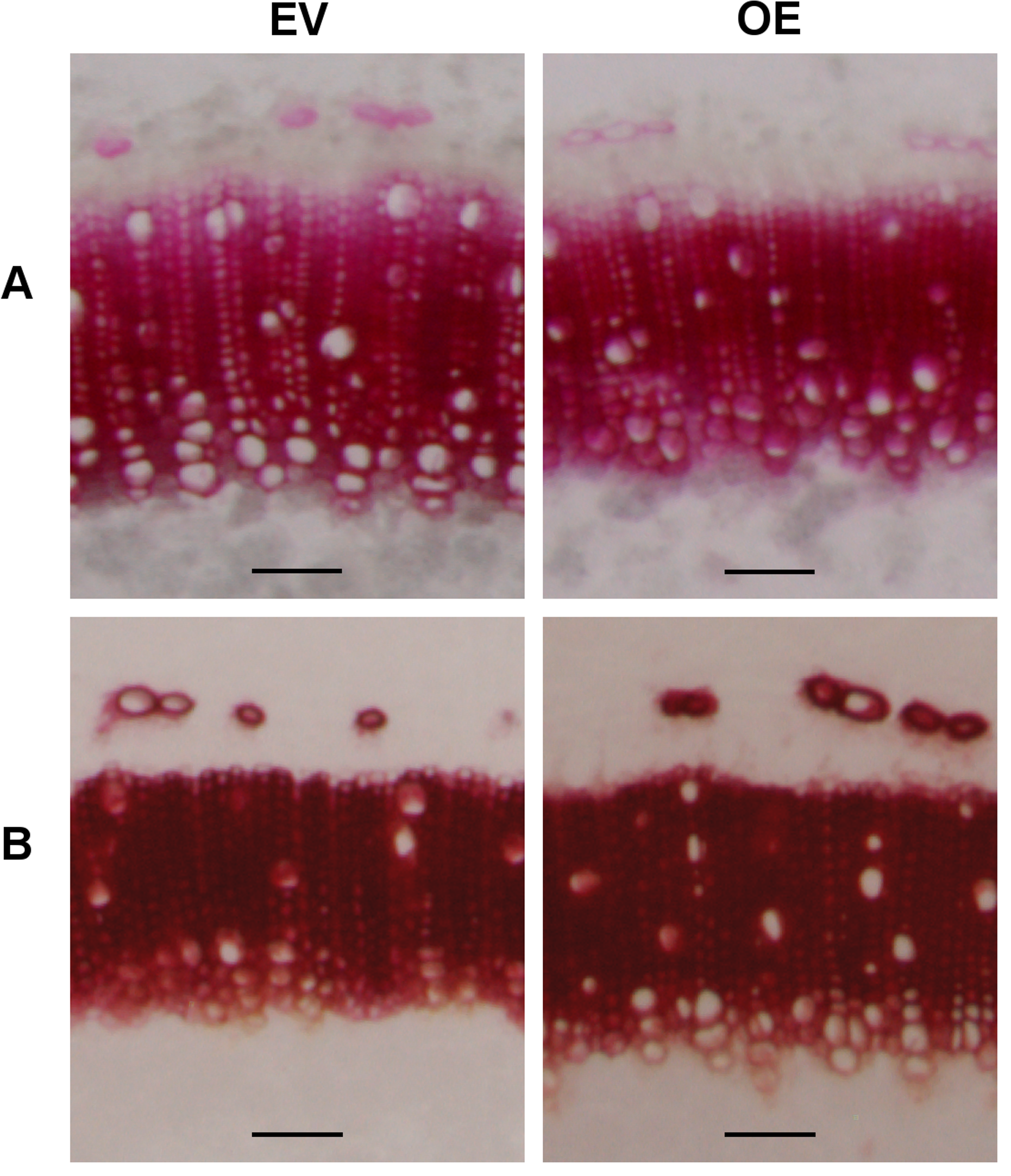

Supplement: Supplementary file 5 [file Image_3.TIF]
